# Effect of Apitherapy Formulations against Carbon Tetrachloride-Induced Toxicity in Wistar Rats after Three Weeks of Treatment

**DOI:** 10.3390/molecules190913374

**Published:** 2014-08-29

**Authors:** Calin Vasile Andritoiu, Lacramioara Ochiuz, Vasile Andritoiu, Marcel Popa

**Affiliations:** 1 Apitherapy Medical Center, Balanesti, Nr. 336-337, Gorj 217036, Romania; E-Mails: dr_calin_andritoiu@yahoo.com (C.V.A.); apitherapy2003@yahoo.com (V.A.); 2 Gheorghe Asachi Technical University of Iasi, Department of Natural and Synthetic Polymers, Iasi 700050, Romania; E-Mail: marpopa2001@yahoo.fr; 3 Vasile Goldis Western University of Arad, Liviu Rebreanu Street, 86, Arad 310045, Romania; 4 Grigore T. Popa University of Medicine and Pharmacy of Iasi, Department of Pharmaceutical Technology, Faculty of Pharmacy, Universitatii Street, 16, Iasi 700115, Romania

**Keywords:** carbon tetrachloride, hepatotoxicity, apitherapy diet, hepatoprotection

## Abstract

The human body is exposed nowadays to increasing attacks by toxic compounds in polluted air, industrially processed foods, alcohol and drug consumption that increase liver toxicity, leading to more and more severe cases of hepatic disorders. The present paper aims to evaluate the influence of the apitherapy diet in Wistar rats with carbon tetrachloride-induced hepatotoxicity, by analyzing the biochemical determinations (enzymatic, lipid and protein profiles, coagulation parameters, minerals, blood count parameters, bilirubin levels) and histopathological changes at the level of liver, spleen and pancreas. The experiment was carried out on six groups of male Wistar rats. Hepatic lesions were induced by intraperitoneal injection of carbon tetrachloride (dissolved in paraffin oil, 10% solution). Two mL per 100 g were administered, every 2 days, for 2 weeks. Hepatoprotection was achieved with two apitherapy diet formulations containing honey, pollen, propolis, *Apilarnil*, with/without royal jelly. Biochemical results reveal that the two apitherapy diet formulations have a positive effect on improving the enzymatic, lipid, and protein profiles, coagulation, mineral and blood count parameters and bilirubin levels. The histopathological results demonstrate the benefits of the two apitherapy diet formulations on reducing toxicity at the level of liver, spleen and pancreas in laboratory animals.

## 1. Introduction

Carbon tetrachloride (CCl_4_) is an industrial solvent and a xenobiotic used to induce chemical hepatic injuries in laboratory animals. The CCl_4_-induced hepatic lesions are a common experimental model for the screening of the hepatoprotective activity of certain drugs, CCl_4_ being a strong hepatotoxic agent and a single exposure to it rapidly leading to severe hepatic necrosis and steatosis [1,2,3]. CCl_4_ is metabolized by the hepatic microsomal P_450_ isoenzymes through the reductive dehalogenation of the toxic in the endoplasmic reticulum of hepatic cells resulting in the unstable free radicals of trichloromethyl (CCl_3_·) and trichloromethyl peroxyl (CCl_3_O_2_·).

It has been demonstrated that propolis has a hepatoprotective potential, as well as anti-inflammatory, immunostimulating, antiviral, and antibacterial effects [4,5,6,7]. Lin *et al*. revealed that ethanolic propolis extract significantly prevents the increase of the levels of microsomal enzymes and the lipidic peroxidation in rats that were administered alcohol [8]. The hepatoprotective effect of royal jelly (RJ) in CCl_4_-induced liver damage has been reported by Cemek *et al*. [9]. Laboratory animals that were administered this toxic substance presented histopathological modifications in the liver and biochemical alterations. Bee pollen is a very important source of saccharides, lipids, proteins and amino acids, vitamins and polyphenolic compounds, mainly flavonoids, thus being able to exert hepatoprotective effects [10,11]. The hepatoprotective effects of honey have also been studied over time [12]. Honey has a long history of use as a natural source of sugars and it is also an important ingredient in traditional medicine due to its antimicrobial and anti-inflammatory properties [13,14].

Many people suffer nowadays from hepatic disorders induced by alcohol consumption, exposure to chemical substances and infections. As chronic and acute hepatic affections continue to cause severe health problems all over the world, numerous experimental studies have taken considerable interest in the role of natural products such as extract of *Coriandrum sativum* [15], root of *Taraxacum officinale* [16], aqueous extract of *Coptidis rhizoma* [17], extract of *Pracparatum mungo* (*Phaseolus mungo* L.) [18], sea buckthorn seed oil (*Hippophae rhamnoides* L., *Elaeagnaceae*) [19] and the alga *Laminaria japonica* [20] in treating hepatic disorders.

The present experiment follows the same direction of research, by investigating the protective effects of two apitherapy diet formulations containing a mixture of propolis, pollen, *Apilarnil*, and honey, with/without royal jelly, against carbon tetrachloride-induced toxicity in Wistar rats. The novelty value is given by the correlation established between the pathological changes in the enzymatic, lipid and protein profiles, the coagulation and blood count parameters, minerals, bilirubin, and the histopathological changes at the level of the liver, pancreas and spleen. The pancreatic and spleen tissues were included in our analysis because we intended to extend our assessment of the effect of CCl_4_ on these tissues, with the understanding that the toxic effects may also occur at other levels than the liver.

## 2. Results

### 2.1. The Content in Total Polyphenol and Flavonoids of Apitherapy Formulations

TPC was expressed in mg/100 g apitherapy formulations and represents the mean value for three determinations for each sample (Table 1).

**Table 1:** Total Polyphenol Content in apitherapy formulations.

Samples	Mean TPC (mg/100 g Formulation)
Formulation I	6.3
Formulation II	6.2

Total content in flavonoids was expressed in mg/100 g apitherapy formulations and represents the mean value for three determinations for each sample (Table 2).

**Table 2:** Total flavonoids content in apitherapy formulations.

Samples	Mean Flavonoids (mg/100 g Formulation)
Formulation I	8.7
Formulation II	8.55

### 2.2. Biochemical Analysis

#### 2.2.1. Enzymatic Profile

Regarding the results for the enzymatic profile, a significant increase of AST, ALT, ALP, and GGT can be observed in animals with CCl_4_ induced hepatopathy (group IV) when compared to the control group fed with standard food (group I) and control groups which were given the apitherapy diet/apitherapy diet and RJ (groups II and III), thus demonstrating the hepatotoxic potential of CCl_4_. The results presented in the below tables contain the significance from the statistical point of view, defined by one of the letters (a), (b), (c), (d) meaning: (a, *p* < 0.05 *vs.* group I) the value is statistically significant in comparison with group I (control group standard food); (b, *p* < 0.0001 *vs.* group II) the value is statistically significant in comparison with group II (control group apitherapy diet); (c, *p* < 0.0001 *vs.* group III) the value is statistically significant in comparison with group III (control group apitherapy diet + RJ); (d, *p* < 0.0001 *vs.* group IV) the value is statistically significant in comparison with group IV (CCl_4_ group). If the obtained value was statistically significant in comparison with more than one of the experimental groups, the corresponding letters are given.

Administration of apitherapy diet (formulation I) to animals with CCl_4_ induced hepatopathy (group V) results in the decrease of the values for the hepatic enzymes compared to the CCl_4_ group (group IV) that received only standard food, revealing the hepatoprotective effect of the tested apitherapy diet formulation. Furthermore, administration of the apitherapy diet and RJ (formulation II) to animals with CCl_4_ induced hepatotoxicity (group VI) determines the improvement of the values for the liver enzymes compared to the untreated group (group IV), also demonstrating the positive effect of RJ (Table 3).

**Table 3:** Enzymatic profile results.

Groups	Enzymatic Parameters			
	AST (UI/L)	ALT (UI/L)	GGT (UI/L)	ALP (UI/L)
Group I	152 ± 31.33	66.82 ± 27.43	0.58 ± 0.03	107.57 ± 21.6
Group II	67.571 ± 30.69 ^a^	42.45 ± 8.75	0.54 ± 0.07	102 ± 16.01
Group III	52.285 ± 9.25 ^a^	35.35 ± 4.5 ^a^	0.45 ± 0.05	98.8 ± 16.3
Group IV	385.1 ± 44.95 ^a,b,c^	99.33 ± 21.51 ^a,b,c^	0.95 ± 0.34 ^b,c^	170.4 ± 14.82 ^a,b,c^
Group V	93.7 ± 13.75 ^a,d^	51.81 ± 13.72 ^d^	0.55 ± 0.36 ^d^	110.9 ± 26.33 ^d^
Group VI	73.4 ± 3.59 ^a,d^	49.77 ± 6.89 ^d^	0.28 ± 0.27 ^d^	112.3 ± 21.82 ^d^

#### 2.2.2. Lipidic Profile

Regarding the lipid profile, the present experiment notes that the administration of the apitherapy diet (formulation I or formulation II) to laboratory animals with CCl_4_ induced toxicity determines the decrease of lipid profile values (total cholesterol, triglycerides, VLDL) when compared to CCl_4_ group (Table 4).

**Table 4:** Serum lipid profiless.

Groups	Serum Lipid Values			
	TC (mg/dL)	VLDL (mg/dL)	TG (mg/dL)	HDL (mg/dL)
Group I	72 ± 14.32	14.14 ± 7.08	47.14 ± 20.95	50.17 ± 7.74
Group II	62.71 ± 7.34	18.71 ± 9.06	82.42 ± 16.64	52.5 ± 7.59
Group III	55.57 ± 9.36 ^a^	23.71 ± 3.25 ^a^	114 ± 20.24 ^a^	53.3 ± 6.87
Group IV	95.3 ± 11.83 ^a,b,c^	36 ± 7.03 ^a,b,c^	181.2 ± 35.24 ^a,b,c^	36.64 ± 3.4 ^a,b,c^
Group V	59.6 ± 7.33 ^d^	17 ± 3.74 ^d^	84.5 ± 18.42 ^a,d^	50.45 ± 6.72 ^d^
Group VI	57.6 ± 5.14 ^a,d^	16.2 ± 3.04 ^d^	81.4 ± 15.95 ^a,d^	54.45 ± 6.68 ^d^

It is also worth mentioning that the administration of formulation II to laboratory animals with CCl_4_ induced toxicity leads to superior results in comparison with the group treated with formulation I, bringing us to the conclusion that RJ has an important therapeutic value in this direction. Future fundamental and clinical studies are needed to establish the mechanisms through which apitherapy formulations get involved in modulating the lipid profile in the affection of the liver.

#### 2.2.3. Protein Profile

Regarding the protein profile, in animals with CCl_4_ induced hepatopathy, the following can be noticed: the decrease of total proteins, the decrease of albuminemia, the decrease of the albumin/globulin ratio, and the increase of the globulins when compared with the control groups fed with standard food, apitherapy diet, apitherapy diet and RJ, respectively.

The administration of the apitherapy diet (formulations I and II) to animals with CCl_4_ induced hepatopathy, when compared to the CCl_4_ group, results in: the increase of the serum levels of total proteins, the increase of the albuminemia, the decrease of the globulin values, and the increase of the albumin/globumin ratio (Table 5).

**Table 5:** Total serum protein profile.

Groups	Serum Protein Values							
	TP	ALB	GLO	ALPHA-1	ALPHA-2	BETA	GAMMA	A/G
Group I	6.13 ± 0.25	29.71 ± 0.75	70.28 ± 0.75	28.87 ± 0.59	4.57 ± 1.32	25.37 ± 3.36	10.02 ± 0.37	0.42 ± 0.01
Group II	7.02 ± 0.5 ^a^	40.84 ± 2.36 ^a^	59.3 ± 2.13 ^a^	24.54 ± 1.88 ^a^	5.05 ± 0.45	20.22 ± 1.72 ^a^	9.32 ± 0.77	0.69 ± 0.0 ^a^
Group III	7.33 ± 0.42 ^a^	40 ± 1.41 ^a^	60.28 ± 1.25 ^a^	25.85 ± 1.32 ^a^	6.42 ± 0.31 ^a^	18.6 ± 0.33 ^a^	9.51 ± 0.19	0.65 ± 0.0 ^a^
Group IV	5.27 ± 0.53 ^a,b,c^	15.1 ± 3.28 ^a,b,c^	84.5 ± 3.24 ^a,b,c^	29.04 ± 0.69 ^b,c^	7.3 ± 0.8 ^a,b^	27.32 ± 1.14 ^b,c^	20.88 ± 1.79 ^a,b,c^	0.17 ± 0.0 ^a,b,c^
Group V	6.92 ± 0.46 ^a,d^	40.12 ± 3.39 ^a,d^	59.9 ± 3.37 ^a,d^	22.77 ± 3.04 ^a,c,d^	7.85 ± 1.53 ^a,b,c^	22.17 ± 3.13 ^a,c,d^	7.21 ± 1.39 ^a,b,c,d^	0.67 ± 0.08 ^a,d^
Group VI	7.13 ± 0.43 ^a,d^	41.49 ± 1.41 ^a,d^	58.51 ± 1.41 ^a,d^	23.75 ± 2.05 ^a,d^	6.3 ± 0.5 ^a^	18.76 ± 0.56 ^a,c,d^	9.46 ± 0.45 ^d,e^	0.7 ± 0.04 ^a,d^

**Table 6:** Coagulation parameters and minerals results.

Groups	Coagulation Parameters				Minerals			
	PT Seconds	TT Seconds	F mg/dL	INR	Fe μg/dL	K mmol/L	iCa mg/dL	sCa mg/dL
Group I	18.14 ± 1.01	21.65 ± 0.89	322 ± 8.64	1.52 ± 0.09	56 ± 4.83	5.15 ± 0.27	4.19 ± 0.13	8.81 ± 0.96
Group II	16.5 ± 0.36	18.64 ± 1.38	214.2 ± 9.01 ^a^	1.36 ± 0.02	69.71 ± 9.01	5.28 ± 0.3	4.42 ± 0.21	9.78 ± 0.5 ^a^
Group III	15.65 ± 0.39 ^a^	19.04 ± 1.18	208.4 ± 5.79 ^a^	1.29 ± 0.03	94 ± 7.91	5.32 ± 0.22	4.58 ± 0.16	10.5 ± 0.39 ^a,b^
Group IV	19.51 ± 1.8 ^b,c^	31.12 ± 4.49 ^a,b,c^	385 ± 48.95 ^a,b,c^	1.61 ± 0.15	247.8 ± 77.4 ^a,b,c^	5.63 ± 0.46	3.54 ± 0.49 ^a,b,c^	7.83 ± 0.4 ^a,c^
Group V	18.18 ± 1.28 ^b,c^	24.42 ± 2.7 ^b,c,d^	267.1 ± 56.83 ^d^	1.57 ± 0.13	173.1 ± 69.27 ^a,b,c,d^	5.21 ± 0.56	4.2 ± 0.1 ^d^	9.58 ± 0.49 ^c,d^
Group VI	17.68 ± 0.87 ^c,d^	24.16 ± 1.31 ^b,c,d^	241.2 ± 47.92 ^a,d^	1.49 ± 0.08	124.5 ± 32.19 ^d^	5.25 ± 0.21	4.38 ± 0.15 ^d^	10.15 ± 0.6 ^a,d^

#### 2.2.4. Coagulation Parameters

In animals with CCl_4_ induced hepatopathy (group IV) we could observe the increase of Quick’s time (PT), thrombin time (TT) and fibrinogen (F). Administration of the apitherapy diet (formulation I) to laboratory animals with CCl_4_ induced hepatopathy (group V) and also the administration of apitherapy diet + RJ (formulation II) to group VI leads to normal values of PT and TT, values that can be compared to those of the animals without hepatopathy (Table 6).

#### 2.2.5. Minerals

Administration of the apitherapy diet (formulation I) to laboratory animals with CCl_4_ induced hepatopathy (group V) determines, when compared to the CCl_4_ group (group IV), the decrease of iron levels and increase of the ionized and serum calcium, modifications also observed for the animals with CCl_4_ induced hepatopathy that were treated with formulation II (apitherapy diet + RJ) (Table 6).

#### 2.2.6. Blood Count Parameters

In animals with CCl_4_-induced hepatopathy (group IV) we could see the increase of RDW, the decrease of RBC, HCT and Hgb, and the decrease of hemoglobin. Administration of apitherapy diet (formulation I) and apitherapy diet + RJ (formulation II) to the animals with CCl_4_ induced hepatopathy leads to a normalization of the blood count parameters (Table 7).

**Table 7:** Complete blood count parameters results.

Groups	Blood count															
	WBC (10^3^/mcL)	RBC (10^3^/mcL)	Hgb (g/dL)	HCT (%)	MCV (fL/Cell)	MCH (pg/Cell)	MCHC (g/dL)	RDW (%)
Group I	5.87 ± 1.62	8.51 ± 0.04	14.71 ± 0.64	44.65 ± 2.05	53.11 ± 0.83	16.88 ± 1.29	32.91 ± 0.65	18.74 ± 0.21
Group II	10.78 ± 3.12 ^a^	7.53 ± 0.61	14.48 ± 1.49	44.25 ± 3.24	58.78 ± 2.01 ^a^	18.97 ± 0.83 ^a^	32.41 ± 1.15	16.38 ± 1.04
Group III	11.92 ± 1.07 ^a^	7.71 ± 0.24	15.02 ± 0.91	45.71 ± 1.28	59.38 ± 1.25 ^a^	19.44 ± 0.85 ^a^	32.78 ± 1.15	16.42 ± 0.57
Group IV	4.86 ± 0.69 ^b,c^	7.22 ± 1.21 ^a^	13.27 ± 1.47 ^a^	39.9 ± 4.3 ^a,b,c^	56.61 ± 5.04	18.52 ± 1.78	31.94 ± 0.77	19.51 ± 1.62
Group V	10.39 ± 2.6 ^a,d^	7.68 ± 0.59	14.46 ± 1.16	44.01 ± 2.44 ^d^	57.34 ± 2.67	18.79 ± 0.77 ^a^	32.83 ± 1.0	15.55 ± 1.16
Group VI	11.49 ± 1.01 ^a,d^	7.82 ± 0.22	14.72 ± 0.84	44.16 ± 0.54 ^d^	56.82 ± 3.24	18.89 ± 1.05 ^a^	33.31 ± 0.11 ^d^	15.44 ± 0.9
	**Neu **(**%**)	**Neu **(**10^3^/mcL**)	**Ly **(**%**)	**Ly **(**10^3^/mcL**)	**Mo **(**%**)	**Mo **(**10^3^/mcL**)	**Eo **(**%**)	**Eo **(**10^3^/mcL**)
Group I	27.14 ± 4.3	1.79 ± 0.05	51.48 ± 5.96	3.49 ± 1.09	8.21 ± 0.84	0.53 ± 0.15	8.61 ± 0.57 ^a^	0.431 ± 0.11
Group II	12.94 ± 3.6 ^a^	0.79 ± 0.13 ^a^	75.95 ± 4.62 ^a^	5.27 ± 0.65	3.61 ± 0.22 ^a^	0.24 ± 0.02	2.74 ± 0.32 ^a^	0.158 ± 0.02 ^a^
Group III	11.54 ± 1.56 ^a^	1.49 ± 0.03 ^a,b^	74.71 ± 3.19 ^a^	8.74 ± 0.25 ^a,b^	2.40 ± 0.33 ^a^	0.26 ± 0.03	2.7 ± 0.18 ^a^	0.30 ± 0.03
Group IV	19.34 ± 2.04 ^a,b,c^	1.00 ± 0.13 ^a,b^	69 ± 3.14 ^a,b^	3.64 ± 0.55 ^c^	7.71 ± 2.49 ^b,c^	0.30 ± 0.06	3.89 ± 1.74 ^a^	0.28 ± 0.09 ^a,b^
Group V	12.61 ± 3.45 ^a,d^	1.08 ± 0.36 ^a,c^	76.72 ± 3.74 ^a,d^	6.52 ± 1.92 ^a,c,d^	3.15 ± 0.56 ^a,d^	0.24 ± 0.03	2.76 ± 0.27 ^a^	0.23 ± 0.1 ^a^
Group VI	14.98 ± 1.72 ^a,d^	1.34 ± 0.25 ^b,d^	77.61 ± 2.71 ^a,d^	8.17 ± 1.24 ^a,b,d^	2.95 ± 0.74 ^a,d^	0.53 ± 0.83	3.24 ± 0.7 ^a^	0.25 ± 0.07 ^a,b^

#### 2.2.7. Bilirubin

In animals with CCl_4_-induced hepatopathy (group IV), when compared with the other experimental groups, the increase of total and indirect bilirubin can be noticed. Administration of the two apitherapy formulations (formulation I–group V, and formulation II-group VI) leads to the decrease of the above mentioned parameters (Table 8).

**Table 8:** Bilirubin values.

Groups	TB (mg/dL)	DB (mg/dL)	IB (mg/dL)
Group I	0.105 ± 0.02	0.014 ± 0.005	0.091 ± 0.02
Group II	0.121 ± 0.006 ^b^	0.011 ± 0.003	0.11 ± 0.008
Group III	0.087 ± 0.004	0.01	0.077 ± 0.004 ^b^
Group IV	0.135 ± 0.01 ^a,c^	0.01	0.125 ± 0.01 ^a,c^
Group V	0.121 ± 0.02 ^d^	0.01	0.111 ± 0.02 ^c^
Group VI	0.113 ± 0.02	0.013 ± 0.004	0.1 ± 0.02

### 2.3. Histopathological Examination

#### 2.3.1. Histopatological Evaluation of Liver Samples

**Figure 1:**
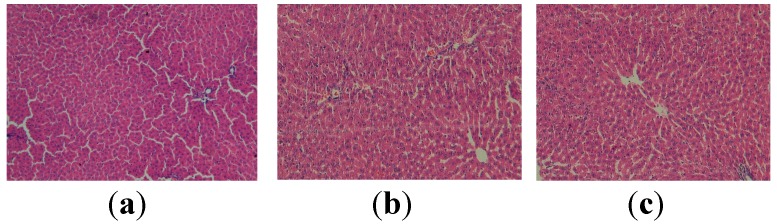
Histopathological evaluation of the liver tissue for the control groups. (**a**) Normal aspect (group I, control group-standard food) (HEx100); (**b**) Normal aspect (group II, control group-apitherapy diet) (HEx100); (**c**) Normal aspect (group III, control group-apitherapy diet + royal jelly) (HEx100).

The micrographs shown in Figure 1 illustrate the normal aspect of the hepatic liver for the control groups fed with standard food, with apitherapy diet (formulation I) and with apitherapy diet and RJ (formulation II), respectively.

**Figure 2:**
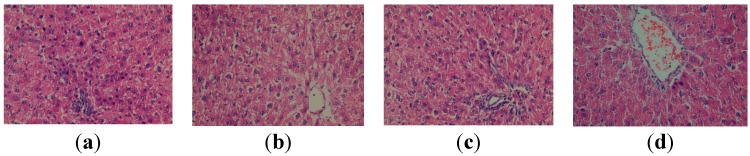
Histopathological evaluation of the liver tissue for group IV (CCl_4_ group): (**a**) Inflammatory infiltrates (HEx400); (**b**) Intralobular cytolysis (HEx400); (**c**) Apoptotic cells, Kupffer cell hyperplasia (HEx400); (**d**) Centrilobular stasis (HEx400).

At the level of the hepatic tissue of animals with CCl_4_-induced hepatopathy, the following can be seen: portal and intralobular inflammatory infiltrates, apoptotic cells, numerous intralobular cytolysis centres, Kupffer cell hyperplasia (Figure 2). The administration of the apitherapy diet (formulation I) in laboratory animals with CCl_4_ induced hepatopathy (group V) reveals the improvement of the histoarchitectural changes of the liver towards the normal aspect, showing only isolated vesicular steatosis, discrete inflammatory infiltrate and discrete congestion.

**Figure 3:**
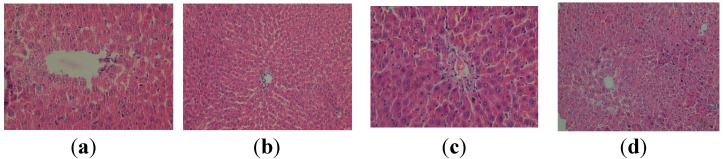
Histopathological evaluation of the liver tissue for the treated groups: (**a**) *group V* (group CCl4-apitherapy diet) Isolated vesicular steatosis (HEx400); (**b**) Discrete inflammatory infiltrate (HEx200); (**c**) Discrete congestion (HEx400); *group VI* (group CCl4-apitherapy diet + RJ) (**d**) Discrete inflammatory infiltrate (HEx200).

This demonstrates the positive influence of the apitherapy treatment upon the histoarchitecture of the liver affected by the administration of CCl_4_ (Figure 3). Even better results can be observed subsequently to the administration of the apitherapy diet and RJ (formulation II), where only a discrete inflammatory infiltrate is present (Figure 3).

#### 2.3.2. Histopatological Evaluation of Pancreas Samples

**Figure 4:**
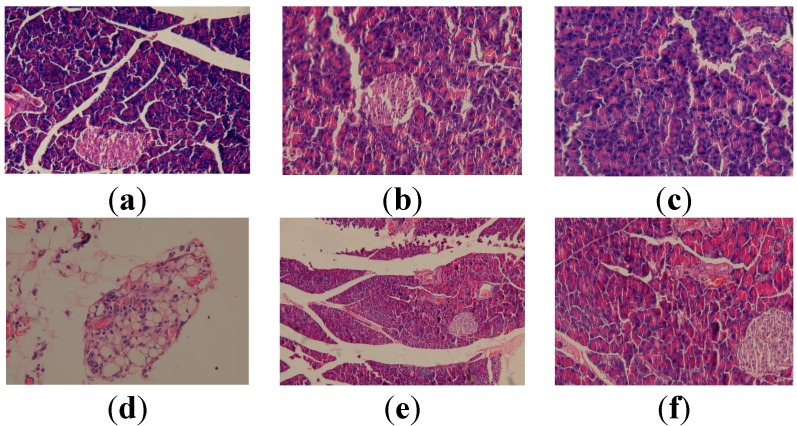
Histopathological evaluation of pancreatic tissue for the experimental groups. (**a**) Normal aspect (group I, control group-standard food) (HEx100); (**b**) Normal aspect (group II, control group-apitherapy diet) (HEx100); (**c**) Normal aspect (group III, control group-apitherapy diet + RJ) (HEx200); (**d**) Liponecrosis (group IV, CCl_4_ group-untreated) (HEx400); (**e**) Normal aspect (group V, CCl_4_ group + apitherapy diet). (HEx100); (**f**) Normal aspect (group VI, CCl_4_ group-apitherapy diet + RJ). (HEx100).

The micrographs obtained from the fragments of pancreas of the animals that were not exposed to CCl_4_, but were given standard food (group I), apitherapy diet–formulation I (group II), apitherapy diet and RJ–formulation II (group III) are presented in Figure 4, revealing a normal aspect of the pancreas, without pathological modifications. 

In animals chronically exposed to CCl_4_, unequal islets of Langerhans can be observed at the level of the pancreatic tissue, some of them hypertrophied, while in the peripancreatic adipose tissue, liponecrosis is present (Figure 4).

Administration of the apitherapy diet/apitherapy diet and royal jelly to animals with CCl_4_ induced hepatotoxicity reveals an improvement of the pancreatic histoarchitecture, although liponecrosis can be noticed in the peripancreatic adipose tissue (Figure 4).

#### 2.3.3. Histopatological Evaluation of Splenic Tissue

**Figure 5:**
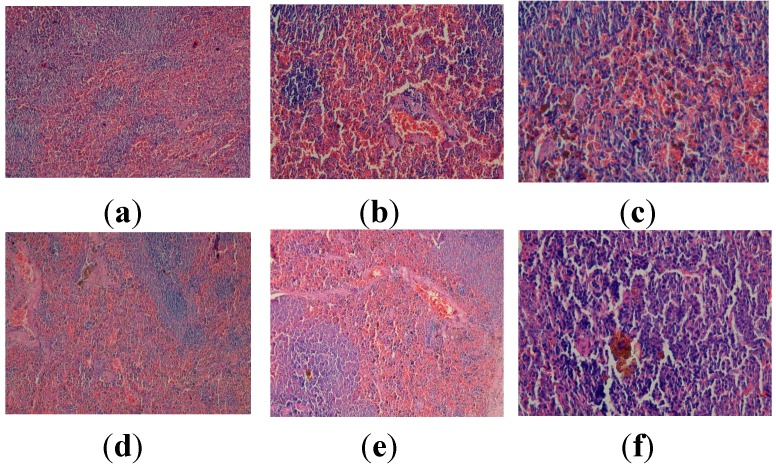
Histopathological evaluation of splenic tissue for the experimental groups. (**a**) Normal aspect (control group-standard food) (HEx100); (**b**) Dilated sinusoids (CCl_4_ group) (HEx200); (**c**) Stasis in red pulp, pigment (CCl_4_ group) (HEx400); (**d**) red pulp with dilated sinusoids (CCl_4_ group-apitherapy diet) (HEx100); (**e**) Stasis and hemosiderin pigment in red pulp (CCl_4_ group-apitherapy diet + RJ) (HEx100); (**f**) Pigment in red pulp (CCl_4_ group-apitherapy diet + RJ) (HEx400).

In animals with CCl_4_ induced toxicity (group IV), at the level of splenic tissue the following can be noticed: red pulp with dilated sinusoids filled with blood, marked vasodilation and stasis of the red pulp, presence of blood pigments (Figure 5).

After a three-week experiment, the benefic impact of the treatment with apitherapy diet/apitherapy diet and RJ is not as important as in the case of the hepatic tissue. Thus, the administration of apitherapy diet (formulation I) to laboratory animals with CCl_4_ induced hepatopathy (group V) determines, at the level of splenic tissue, the following aspects: red pulp with dilated sinusoids, rarely filled with blood (Figure 5). The administration of apitherapy and RJ (formulation II) to laboratory animals with CCl_4_ induced hepatopathy (group VI) reveals important stasis with blood pigment in red pulp, and also blood pigment in white pulp (Figure 5).

## 3. Discussion

The results of the present experiment are in agreement with results obtained in other experimental studies [21,22,23,24]. For example, the study of Cemek *et al*.demonstrates that the administration of CCl_4_ leads to severe acute liver affection in rats, revealed by significant increase of AST and ALT serum levels. The same study reveals that the treatment with RJ (included in the present experimental study in formulation II) remarkably counteracts the severe acute hepatic affection induced by CCl_4_, which can be seen in the decrease of the serum activity of AST and ALT. Furthermore, the histopathological evaluation shows that the hepatic lesions induced by CCl_4_ improve after treatment with RJ [9]. 

Propolis, an ingredient in the two formulations administered in the present experiment, is known to improve the enzymatic profile. This result may be explained by the rapid regeneration of the parenchyma cells produced by the presence of the bioactive substances in propolis, such as flavonoids and their esters, mainly CAPE (caffeic acid phenethyl ester), which prevent the membranous fragility and subsequently decrease the levels of enzymatic markers in blood circulation [25,26].

The most important result obtained in the present experiment is the increase of HDL, following both the administration of formulation I and formulation II to laboratory animals with CCl_4_ induced toxicity. The positive effect of the administered apitherapy diet upon the lipid profile is based on the pharmacological action exerted by the ingredients in these preparations: honey, propolis, pollen, and RJ.

Thus, an experimental study regarding honey has demonstrated that the ingestion of this bee product has a protective effect upon the cardiovascular system by reducing blood cholesterol levels, LDL and triglycerides and by producing a slight increase of HDL levels [27].

Propolis therapy has been demonstrated to reduce the high levels of triglycerides, total cholesterol and esters of cholesterol, probably through the antioxidant mechanism exerted by the flavones in propolis [25]. The intensification of the oxidative stress enhances the influence of non-essential fatty acids which, in turn, increase the serum and tissue levels of cholesterol and triglycerides. It has been shown that antioxidants and flavonoids can act as inhibitors of lipid peroxidation by neutralizing the radicals of polyunsaturated fatty acids and by interrupting the chain reactions [28]. Lipid peroxidation is an important biological consequence of cellular oxidative stress and is one of the main causes of hepatic lesions produced by CCl_4_ mediated by the free radicals derived from this toxic substance [3,21,29,30,31].

Regarding the association of RJ in modulating the lipid profile, it has been shown that RJ significantly reduces the values of cholesterol and triglycerides in rabbits [27]. RJ has also demonstrated its physiological and biochemical cardioprotective effect in experiments on mice. It is not known, for the moment, the exact mechanism by which RJ exerts its strong anti-lipid peroxidation activity, but it is considered to be due to its antioxidant potential [9].

The hepatic affection is characterized by the imbalance of the serum levels of total proteins, expressed by the deterioration of the albumin/globulin ratio. The decrease of the albumins level is accompanied by the increase of the globulins level, mainly of γ-globulins (less frequently of α-globulins or β-globulins). The improvement of the albumin/globulin ratio involves the increase of serum albumins, accompanied by a proportional decrease of the globulin values.

The deterioration of the albumin/globulin ratio is constantly accompanied by electrolytic damages, the most important of them being the imbalance of the phosphocalcium metabolism at the level of ionized calcium (hipocalcemia at the level of serum calcium is rarely met). The deterioration of the albumin/globulin ratio in favour of globulins is expressed by the decrease of albumin serum levels with the same amounts as the decrease of ionized calcium. The reduced albumin levels of are associated with reduced ionized calcium levels, even if the total serum calcium levels are normal. During the monitorization of the results after the apitherapy treatment, another recurring situation could be observed: the increase of the albumin levels and rebalance of the albumin/globulin ratio is clearly dependent on and accompanied by the reestablishment of the normal values of the ionized calcium. From the albumin-ionized calcium correlation, the following can be noted: the decrease or increase of the albumin levels are constantly correlated with the decrease or increase of the ionized calcium; the reduced values of albumin and ionized calcium concentrations are found at the beginning of liver damage.

Honey increases the *in vitro* and *in vivo* absorption of calcium in studies on laboratory animals [27]. Propolis apparently increases the absorption and use of different minerals, due to the presence of the derivatives of the organic acids that improve physiological functions by regulating the enzyme-dependent ionic activity. Haro *et al*. demonstrate the benefic effects of pollen and/or propolis on the metabolism of iron, calcium, phosphorus and magnesium in nutritional iron-deficiency anemia in rats [32]. The optimal use of calcium by the organism cannot take place in the following conditions: avitaminosis (including some water-soluble vitamins), demineralization, excess of proteins and sugars, unbalanced diets through excessive intake of sodium, phosphorus, caffeine, foods containing too many vegetal fibers, oxalic acid, phytic acid, as well as the uncontrolled action of free radicals.

In the present experiment, it can be noticed that the administration of apiterapy diet (formulations I and II) leads to normal values of the serum iron and potassium levels, and also of the ionized calcium for the groups that have been given CCl_4_. The biochemical mechanisms involved in acquiring CCl_4_ hepatotoxicity are generated by the peroxidation of lipids produced by trichloromethyl radical (CCl_3_). CCl_4_ is metabolized by cytochrome P_450_ to CCl_3_ radical that induces the peroxidation of membrane lipids and disturbs Ca^2+^ homeostasis thus inducing liver tissue damage [33,34]. Because the two apitherapy formulations have an effect on the normalization of calcium, it can be assumed that they also act on cytochrome P450. Further studies on lipid peroxidation and antioxidant systems will bring additional data into this matter. For now, we may safely assume that the two apitherapy formulations affect lipid peroxidation due to the antioxidant factors present in the composition of bee products and, not lastly, to the vitamin E in honey and pollen, since vitamin E is known to have an effect on lipid peroxidation in intoxication by CCl4 [35].

As for the blood count parameters, the administration of the two apitherapy formulations to the groups with experimentally induced hepatopathy leads to the increase of WBC, the improvement of RBC, Hgb, and RDW values, the increase of HEM to normal levels, the increase of CHEM, the increase of thrombocytres, keeps MPV and PDW within normal range, brings neutrophils to normal values (expressed as % and 10^3^/mcL), and decreases the eosinophils (expressed as % and 10^3^/mcL).

The high values of total bilirubin for the group with CCl_4_ intoxication is correlated with the decrease of RBC in the blood samples of the same group, as the increase of the RBC death is a possible source of bilirubin formation. In bilirubin levels, the administration of the two apitherapy formulations to the groups with experimental induced hepatopathy improves the bilirubin levels, that are albumin dependent. It is known that bilirubin, a hydrophobic and potentially toxic substance, circulates through the plasma bound to albumin [36].Our results show the decrease in the total, direct and indirect bilirubin values in groups with CCl_4_ induced hepatopathy that received the apitherapy treatment when compared to the animals with the same induced affection that received standard food only.

In our experiment, it has been shown that administration of CCl_4_ to Wistar rats produces important histological modifications at the level of the hepatic tissue, but less relevant histopathological changes at the level of spleen and pancreas. 

## 4. Experimental Section

### 4.1. Animal Environment, Housing and Management

The experiment was performed on sixty adult male Wistar rats with a body weight of 220–250 g. The animals were kept in a diffusely lit and temperature-controlled room with a diurnal 12 h light cycle, where the temperature (22 ± 0.5 °C) and relative humidity (65%–70%) were kept constant. The animals were given free access to standard laboratory diet and water before the experiments.

All the experimental proceedings achieved on laboratory animals (Wistar rats) in this study were in agreement with the guidelines of animal bioethics from the Act on Animal Experimentation and Animal Health and Welfare Act from Romania and were in compliance with the European Council Directive of 24 November 1986 (86/609/EEC). The experiment was approved by the Ethics Commission of Grigore T. Popa University of Medicine and Pharmacy of Iasi.

### 4.2. Bee Products

Honey, propolis, *Apilarnil*, and pollen were provided by Stupina LLC, Balanesti, Gorj, while royal jelly was commercially acquired from the market (lyophilized royal jelly produced by The Beekeeping Research and Development Institute, Bucharest, Romania). Chemical composition of propolis: 45%–55% resins and balsams; 7.5% to 35% vegetable waxes and beeswax; 10%–15% volatile essential oils; 5% pollen; 5% phenolic acids and their esters, polyphenols, lignans, sesquiterpene, quinones, steroids and amino acids (pyroglutamic acid, arginine, proline). It also contains hydrosoluble vitamins: B1, B2, B3, B5 and C; liposoluble vitamins: A and E; and minerals Na, K, Mg, Ca, Ba, B, Cr, Zn, Se, Fe, Mn.

Chemical composition of pollen: 3.27%–34.62% reducing sugars (glucose, fructose, lactose, raffinose, stachiloza); 0.5%–20% non-reducing sugars; 1%–20% of fat to fatty acid esters, oleic, linoleic, linolinic acid, palmitic acid, stearic acid and arachidonic; amino acids (5.70% arginine, 2.4% histidine, 4.5% isoleucine, leucine 6.7%, 5.7% lysine, 1.8% methionine, phenylalanine 3.9%, 4% threonine, tryptophan, 1.3%, 5.7% valine); liposoluble vitamins (A, D, E, K); vitamins of group B and vitamin C, and minerals: K, Zn, Mg, Mn, C, Fe.

Chemical composition of *Apilarnil*: 65%–75% water; 25%–35% dry matter content; 12.9% protein; amino acids; carbohydrates: 6%–10% glucose, 3.16% fructose, 0.03% sucrose; 5%–8% lipids; liposoluble vitamins (A, D, E, K) and water-soluble vitamins (B group C); minerals, hormones, particularly somatotropic hormones and steroid hormones; antivirals; xantophylin; choline.

Chemical composition of honey: 0.4%–8% amino acids; 81.3% sugars of which: 7.5% sucrose; 38.19% fructose; 31.28% glucose; 6.89% maltase; other sugars; enzymes; traces of pollen and royal jelly; organic acids; lipids; antibiotic substances grouped under the heading “inhibin”; antigerminative factors; pigments; aromatic compounds, amino acids, minerals, vitamins-B1, B2, B3, B5, B6, B9, B12; vitamin C and liposoluble vitamins: provitamin A; vitamin D; vitamin E; vitamin K; honey enzymes: diastase, invertase, sucrase, zaharaza, catalase, α and β amylase, acid phosphatase, peroxidase, superoxide dismutase (SOD, superoxide oxidoreductase); α and β glucosidase, acetyltransferase, cytochrome oxidase, ascorbic acid oxidase, tyrosinase, monofenol monooxygenase, glycosyl transferases.

### 4.3. Study Design

The experimental model of hepatic lesion was induced by intraperitoneal (i.p.) injection of CCl_4_ (dissolved in paraffin oil, 10% solution). Two mL per 100 g were administered, every 2 days, for 2 weeks. Treatment was provided in the form of two formulations of the apitherapy diet:

- formulation I, consisting of honey, propolis, *Apilarnil*, and pollen granules
- formulation II that contains, besides the ingredients from formulation I, RJ

The two formulations were prepared daily. Formulation I was administered to groups II and V, in doses of 3.535 g/100 g body weight (bw), while formulation II was administered to groups III and VI, in doses of 3.635 g/100 g body weight (Table 9).

**Table 9:** The daily intake of the two apitherapy diet formulations calculated for 100 g bw.

	Bee Products	Formulation I	Formulation II
**1**.	Honey	2.5 g	2.5 g
**2**.	Propolis	0.01 g	0.01 g
**3**.	*Apilarnil*	0.025 g	0.025 g
**4**.	Pollen	1 g	1 g
**5**.	Royal jelly	-	0.1 g
**Total amount**		**3.535 g**	**3.635 g**

The Wistar rats were randomly divided into six groups of 10 animals each, as follows:

- Group I (control group-standard food)-served as control, and was fed with standard food;
- Group II (control group-apitherapy diet)-fed with apitherapy diet (formulation I–3.535 g/100 g bw/day, for 3 weeks);
- Group III (control group-apitherapy diet + RJ)-fed with apitherapy diet and RJ (formulation II, 3.635 g/100 g bw/day, for 3 weeks);
- Group IV (CCl_4_ group)-i.p. administration of 2 mL of 10% paraffin oil solution of CCl_4_ per 100 g, every 2 days, for 2 weeks, and fed with standard food;
- Group V (group CCl_4_-apitherapy diet)-i.p. administration of 2 mL of 10% paraffin oil solution of CCl_4_ per 100 g, every 2 days, for 2 weeks, and fed with apitherapy diet (formulation I–3.535 g/100 g bw/day, for 3 weeks);
- Group VI (group CCl_4_-apitherapy diet + RJ)-i.p. administration of 2 mL of 10% paraffin oil solution of CCl_4_ per 100 g, every 2 days, for 2 weeks, and fed with apitherapy diet and RJ (formulation II–3.635 g/100 g bw/day, for 3 weeks).

The amounts of the two apitherapy diet formulations calculated for each rat were added to a mixture of cereals (oat, barley, rye, and wheat). The diet was administered separately to each rat (housed in separate individual cages), twice a day: in the morning and at noon. In the end, after three weeks of apitherapy treatment, the animals were sacrificed.

### 4.4. Total Polyphenol and Flavonoids Content in the Studied Apitherapy Formulation

#### 4.4.1. Determination of Total Polyphenol Content in the Studied Apitherapy Formulations

TPC was determined by spectrophotometry, with gallic acid as reference, according to the method described by the International Organization for Standardization (ISO) 14502-1 (ISO 14502-1: 2005). Briefly, 1.0 mL of hydroalcoholic/oil propolis extract was transferred to separate tubes containing 5.0 mL of 1/10 dilution of Folin-Ciocalteu reagent in water. Then, 4.0 mL sodium carbonate solution (7.5% w/v) was added. The tubes were then allowed to stand at room temperature for 60 min before absorbance at 765 nm was measured against water. The mean of three readings was used and the total phenolic content was expressed in mg of gallic acid (mg/100 g). The concentration of polyphenols in the samples was derived from a standard curve of gallic acid ranging from 10 to 200 μg/mL (Pearson’s correlation coefficient: *r^2^ =* 0.9998).

#### 4.4.2. Quantitative Determination of the Flavonoids in the Studied Apitherapy Formulations

*Principle of the method*: For the quantitative determination of the flavonoids it was used a spectrophotometric method based on their property to form in the presence of sodium nitrite and aluminum chloride pink complexes whose absorbance is measured at λ = 510 nm.

*Equipment*: UV-VIS Jasco V-550 spectrophotometer; VelpScientifica vortex; DualRange Mettler XS105 analytical balance.

*Reagents*: Aluminum chloride (Chemical Company SA, Bucharest, Romania); Sodium nitrite (Sigma-Aldrich Laborchemikalien GmbH, Seelze, Germany); Sodium hydroxide (Sigma-Aldrich Chemie GmbH, Steinheim, Germany); DMSO (Sigma-Aldrich Laborchemikalien GmbH); (+)-catechin hydrate (Sigma-Aldrich).

*Procedure*: Extracts of apitherapy formulations I and II were dissolved in DMSO in concentrations of 0.5 mg/mL, 2.5 mg/mL and 20 mg/mL. 0.25 mL of each extract solution was added to a mixture of 1.25 mL ultrapure water and 0.075 mL of 5% sodium nitrite. After 6 min, 0.15 mL of 10% aluminum chloride were added. The mixture was stirred vigorously and allowed to stand for 5 min; then 0.5 mL of 1 M sodium hydroxide solution and 0.275 mL ultrapure water were added. The absorbance of the reaction mixture was immediately determined at λ = 510 nm. The concentration of flavonoids in the extract was determined with a standard curve determined by using (+)-catechin hydrate as reference substance. For this purpose, (+)-catechin stock solutions were prepared with the following concentrations: 0,025; 0.05 0.075; 0.1; 0.125; 0.15 and 0.175 mg/mL, which were processed in the same way as the extracts. The concentration of flavonoids in samples was derived from a standard curve of catechin ranging from 20 to 200 μg/mL (Pearson’s correlation coefficient: *r^2^ =* 0.9981).

The results were expressed in equivalents of (+)-catechin [mg of (+)-catechin/100 g extract]. All determinations were made in triplicate. The results are expressed as the mean of three determinations ± standard deviation.

### 4.5. Biochemical Analysis

Anesthesia was achieved with thiopental (dose of 1 mL/100 g from a 0.01% thiopental solution), and blood samples were collected by the punction of the cord (*i*.*e*., the ventricular region) with a Vacuette^®^ system (three types of vacutainers were used for collection, *i*.*e*., vacutainers without anticoagulant for general biochemical tests, vacutainers with Na citrate 0,105M for the samples in which the protein profile parameters were determined and vacutainers with EDTA anticoagulant for determining the blood count. Collected samples were processed by Synevo Laboratory (Iassy, România). Specific references of the procedure technology used 2010 Ref Type: Catalog; Laboratory Corporation of America, San Francisco, CA. Directory of Services and Interpretive Guide. Ref Type: Internet Communication).

Biochemical analysis and hematological tests:

- enzymatic parameters (aspartate aminotransferase-AST, alanine aminotransferase-ALT, alkaline phosphatase-ALP, gamma-glutamyl transpeptidase-GGT),
- lipid profile (total cholesterol-TC, triglyceride-TG, very low density lipoproteins–VLDL, high-density lipoproteins-HDL),
- protein profile (total proteins-TP, albumin-ALB, globulins-GLO, alpha-1 globulins–ALPHA 1, alpha-2 globulins–ALPHA 2, beta globulins-BETA, gamma globulins–GAMMA, and albumin/globulin ratio–A/B),
- coagulation parameters (Quick’s time-PT, thrombin time-TT, fibrinogen-F, International Normalized Ratio-INR),
- minerals (iron-Fe, potassium-K, serum and ionized calcium–sCa and iCa),
- blood count parameters (number of leukocytes–WBC, number of erythrocytes–RBC, hemoglobin–Hgb, hematocrit–HCT, mean corpuscular volume–MCV, mean corpuscular hemoglobin–MCH, mean corpuscular hemoglobin concentration–MCHC, red blood cell distribution width–RDW, number of neutrophils–Neu 103/mcL, percent of neutrophils–Neu%,number of eosinophils–Eo 103/mcL, percent of eosinophils–Eo%, number of lymphocytes–Ly 103/μL, percent of lymphocytes–Ly%, number of monocytes–Mo 103/mcL, percent of monocytes–Mo%)
- bilirubin (total bilirubin-TB, direct bilirubin-DB, indirect bilirubin-IB).

The determination of the values of the investigated parameters was achieved with an automated analyzer (Aeroset, Abbott, Chicago, IL, USA) and commercial kits (Abbott).

### 4.6. Histopathological Examination

When the absence of vital signs (respiratory rate, heart rate, reflexes) was ascertained, the animals were dissected in order to collect the liver, spleen, and pancreas samples for the evaluation of the histopathological modifications.

The collected samples were fixed in 10% buffered formalin for at least 24 h, progressively dehydrated in solutions containing an increasing percentage of ethanol (60%, 80%, 90%, and 98%, v/v), clarified with amylic alcohol, embedded in paraffin under vacuum, sectioned at 5 μm thickness, deparaffinized, and stained with hematoxylin-eosin (HE).

### 4.7. Presentation of Results in Tables and Statistical Data

The statistical interpretation of the results was performed with One-Way ANOVA test and Tukey’s post-hoc test. The results were given as mean ± standard deviation. The value of *p <* 0.05 was considered significant.

## 5. Conclusions

Based on the results obtained in the present experiment, it can be concluded that apitherapy formulations have a positive impact upon the enzymatic, lipid and protein profiles, improve the levels of coagulation parameters, minerals, blood count parameters, bilirubin, and also have a benefic effect on the histoarchitecture of the liver, spleen and pancreas. Thus, apitherapy formulations have a hepatoprotective effect in carbon tetrachloride induced hepatopathy.
